# Clinical and Prognostic Significance of Serum IgG4 in Chronic Periaortitis. An Analysis of 113 Patients

**DOI:** 10.3389/fimmu.2019.00693

**Published:** 2019-04-04

**Authors:** Federica Maritati, Rossana Rocco, Eugenia Accorsi Buttini, Chiara Marvisi, Maria Nicastro, Maria L. Urban, Paride Fenaroli, Francesco Peyronel, Giuseppe D. Benigno, Alessandro A. Palumbo, Domenico Corradi, Giacomo Emmi, Nicolò Pipitone, Alessandra Palmisano, Augusto Vaglio

**Affiliations:** ^1^Nephrology Unit, Parma University Hospital, Parma, Italy; ^2^Radiology Unit, Parma University Hospital, Parma, Italy; ^3^Pathology Unit, Department of Medicine and Surgery, University of Parma, Parma, Italy; ^4^Internal Medicine, University of Firenze, Firenze, Italy; ^5^Rheumatology Unit, Arcispedale S. Maria Nuova, Reggio Emilia, Italy; ^6^Nephrology Unit, Meyer Children's Hospital, Firenze, Italy; ^7^Department of Biomedical, Clinical and Experimental Sciences, University of Firenze, Firenze, Italy

**Keywords:** periaortitis, IgG4, IgG4-related disease, retroperitoneal fibrosis, hydronephrosis, fibro-inflammatory disorder

## Abstract

**Objective:** Chronic periaortitis (CP) is a rare fibro-inflammatory disorder that incorporates idiopathic retroperitoneal fibrosis, inflammatory abdominal aortic aneurysms, and perianeurysmal retroperitoneal fibrosis. CP is included in the spectrum of IgG4-related disease. Since CP patients rarely undergo diagnostic biopsies, serum IgG4 levels are often used to classify CP as IgG4-related. However, the clinical and prognostic significance of serum IgG4 in CP is unknown.

**Methods:** We measured serum IgG4 in active CP patients and compared the clinical characteristics, response to therapy and outcome of patients with high and normal levels. We also tested the diagnostic significance of IgG4 by comparing its levels in CP patients, healthy and disease controls (malignancies, Erdheim-Chester disease, large-, and small-vessel vasculitis).

**Results:** We studied 113 consecutive patients with active CP. Twenty-four (21.2%) had high serum IgG4 (>135 mg/dL). The demographic, laboratory, and clinical characteristics of patients with high and normal IgG4 were similar, and so were the rates of ureteral obstruction and the disease characteristics on CT, MRI, and ^18^F-FDG-PET. Patients with high IgG4 only had a higher frequency of extra-retroperitoneal fibro-inflammatory lesions (*p* = 0.005). There were no significant differences in response to therapy and relapses between the two groups. Serum IgG4 levels did not discriminate CP from controls.

**Conclusions:** Serum IgG4 levels are high in a minority of CP patients and do not identify specific clinical or prognostic subgroups; only a higher frequency of extra-retroperitoneal lesions is found in high-IgG4 patients. Serum IgG4 levels do not help in the differential diagnosis between CP and its mimics.

## Introduction

Chronic periaortitis (CP) is a rare disease characterized by a peri-aortic and peri-iliac fibro-inflammatory tissue that frequently entraps retroperitoneal structures such as the ureters, causing ureteral obstruction and renal failure. CP includes non-aneurysmal [idiopathic retroperitoneal fibrosis (IRF)] and aneurysmal forms [inflammatory abdominal aortic aneurysms (IAAAs), perianeurysmal retroperitoneal fibrosis (PRF)] (1–3). It may be isolated or develop in the setting of systemic immune-mediated diseases, such as small-vessel vasculitis, systemic lupus erythematosus and rheumatoid arthritis, and is also frequently associated with organ-specific autoimmune diseases such as Hashimoto's thyroiditis (1, 4–6).

CP can also arise in the context of a systemic fibro-inflammatory condition known as IgG4-related disease (IgG4-RD) (7), whose clinical spectrum encompasses sclerosing pancreatitis and cholangitis, chronic sialoadenitis, fibrosing mediastinitis, orbital pseudotumor, tubulointerstitial nephritis, and other conditions (8–10). The pathology of CP is hallmarked by abundant fibrosis and a chronic inflammatory infiltrate rich in lymphocytes, macrophages, plasma cells, and eosinophils. These histological aspects are shared by IgG4-RD, whose peculiarities include the presence of a high proportion of IgG4+ plasma cells (usually >40% of IgG+ plasma cells), obliterative phlebitis, storiform fibrosis, lymphoid follicles, and other features. Therefore, in CP and other conditions belonging to the spectrum of IgG4-RD, a continuum of lesions may be observed, ranging from IgG4-unrelated to full-blown IgG4-related forms.

Previous studies have demonstrated that, based on histological findings, up to 50% of CP cases can be classified as IgG4-related (10–16). No significant differences were found between IgG4-related and -unrelated CP in terms of clinical manifestations, laboratory and imaging features, and rates of obstructive complications (e.g., hydronephrosis). These studies were limited by small sample size, therefore no firm conclusions could be drawn. In addition, most of them used histology and immunohistochemistry to differentiate IgG4-related and—unrelated CP, and although these are the most appropriate tools used to define IgG4-related lesions, it must be acknowledged that periaortic/retroperitoneal biopsy is performed only in a minority of CP patients (usually ~20%) (17, 18) and therefore the distinction between IgG4-related and—unrelated CP must rely on serum IgG4 levels and/or the coexistence of other typical IgG4-related lesions. Finally, only little data is available regarding the response to conventional immunosuppressive therapy and the long-term prognosis of CP based on the IgG4 status.

In this study, we evaluated the IgG4 status (serum levels and, where available, histology and immunohistochemistry) of a large cohort of CP patients at the time of active disease, and compared the clinical presentation, laboratory and imaging findings of IgG4-related and—unrelated cases based on serum levels. Next, we investigated whether serum IgG4 predict response to immunosuppressive therapy and risk of disease relapse. Also, we explored the role of serum IgG4 in the differential diagnosis of CP.

## Patients and Methods

### Patients

In this retrospective observational study we reviewed the clinical records of all patients with CP referred to or diagnosed at our center from January 2010 through June 2017 and investigated the diagnostic, clinical and prognostic significance of serum IgG4. Inclusion criteria were: (i) diagnosis of CP (19); (ii) availability of serum IgG4, measured during active disease (either at first presentation or relapse), before immunosuppressive treatment initiation; (iii) availability of abdominal computed tomography (CT) or magnetic resonance imaging (MRI) at the time of IgG4 measurement. Exclusion criteria were retroperitoneal fibrosis secondary to known causes, such as drugs, infections, and malignancies (19). Drug-related retroperitoneal fibrosis was ruled out after reviewing the patients' medication history for drugs typically associated with the disease (e.g., methysergide, pergolide, ergotamine, methyldopa). Tuberculosis-related forms were screened for using the Quantiferon test and urine cultures for mycobacteria. To screen for underlying tumors, we tested the main neoplastic markers and examined imaging studies, as previously reported (19). The diagnosis of CP was based on CT or MRI findings, on the basis of commonly accepted radiological criteria. Retroperitoneal biopsy was performed only when the mass localization was atypical, when clinical or laboratory findings suggested malignancies or infections, and in patients undergoing surgery (e.g., ureterolysis, aneurysmectomy).

In order to test the significance of serum IgG4 in the differential diagnosis between CP and other conditions, we measured IgG4 in age- and sex-matched healthy controls and in patients affected by aortitis (in the context of Takayasu or giant cell arteritis), Erdheim-Chester disease (ECD), retroperitoneal malignancies (either primary retroperitoneal or metastatic disease), and granulomatosis with polyangiitis (GPA, Wegener's); all of these conditions were included because they can cause aortic or periaortic retroperitoneal inflammation and fibrosis (9, 20, 21). The study was performed in accordance with the declaration of Helsinki and the protocol approved by the ethical committee of Parma. The patients provided informed consent for study participation.

### Data Collection, Radiological Evaluation, and Outcomes

We retrieved data about patient demographics, clinical manifestations, and routine laboratory tests (including serum IgG and IgG subclass levels, blood cell count, renal function, erythrocyte sedimentation rate [ESR], and C-reactive protein [CRP]) performed at the time of active disease (first presentation or relapse). Serum IgG4 were considered high when >135 mg/dL. CT and MRI scans were independently reviewed by two experienced radiologists who were unaware of the patient IgG4 status. The following features were considered when CT and MRI scans were reviewed: localization of the soft-tissue mass, presence of hydronephrosis, aneurysmal aortic dilation, and maximal thickness of the retroperitoneal peri-aortic and/or peri-iliac tissue.

We also analyzed, where available, data regarding treatment and outcome after therapy, including relapses during the post-treatment follow-up. For follow-up imaging studies, measurements (e.g., maximal mass thickness) were performed using the same imaging technique (either CT or MRI) at the same peri-aortic or peri-iliac level chosen for the scans performed at baseline.

Remission was defined as disappearance of disease-related symptoms (e.g., pain, constitutional symptoms), normalization or reduction to <30% of the baseline ESR and CRP levels, and hydronephrosis resolution without need of ureteral stents or nephrostomies; all of the above three criteria had to be met. Relapses were defined as an increase in disease activity represented by recurrent or new symptoms, hydronephrosis, or mass enlargement on CT or MRI (≥20% increase in maximal thickness as compared with previous scans), or by any combination of these.

Finally, where available, we analyzed data regarding ^18^F-fluorodeoxyglucose (FDG)–positron emission tomography (PET) performed at baseline and at the end of therapy, in order to identify any differences between the groups of patients based on the IgG4 status. ^18^F-FDG uptake was graded as follows: 0 = no uptake; 1 = lower than liver uptake; 2 = similar to liver uptake; and 3 = higher than liver uptake. The metabolic responses on FDG-PET were assessed according to PERCIST criteria as: complete (complete resolution of FDG uptake), partial (decline of at least 30% in FDG uptake), no response/stable metabolic disease (neither partial metabolic response nor progressive metabolic disease), or progressive metabolic disease (increase of at least 30% in target lesion activity or appearance of new areas of uptake), as previously described (22).

### Tissue Biopsies

We reviewed the retroperitoneal tissue samples available from CP patients included in the study. The original hematoxylin and eosin-stained slides were evaluated in particular for the presence of the main characteristics of IgG4-RD, such as lymphoplasmacytic infiltrates, storiform fibrosis, obliterative phlebitis, tissue eosinophilia, and lymphoid follicles. In addition, we performed immunohistochemical analyses in order to assess IgG4+ plasma cell infiltration. Unstained retroperitoneal biopsy slides were analyzed with primary antibodies targeting IgG and IgG4 (Abcam, Cambridge, UK, code ab109489, monoclonal, dilution 1:500 and Abcam, code ab109493, monoclonal, dilution 1:800, respectively). In each case, the antigen-antibody reactions were performed in immediately subsequent histological sections in order to explore highly comparable pictures. This immunohistochemical staining was carried out following the manufacturers' protocols; the negative control procedures omitted the primary antibody. The number of IgG4+ and IgG+ plasma cells was assessed in 10 microscopic fields for each of the tested slides using a light microscope (Nikon Eclipse 80i, Tokyo, JPN) at a magnification of 400x (10x eyepiece and 40x lens). The IgG4+/IgG+ plasma cell ratio was calculated dividing the total number of IgG4+ plasma cells by the total number of IgG+ plasma cells, and multiplying this value by 100. The presence of an IgG4+/IgG+ plasma cell ratio >40% was considered indicative of IgG4-related CP (11).

### Statistical Analysis

Continuous variables are presented as median and interquartile range (IQR), while categorical variables as *n* (%). Differences between continuous variables were analyzed using the Mann Whitney test. Categorical variables were compared with Fisher's exact test. Correlations were analyzed using Spearman's rank correlation coefficient. All reported *P*-values are two-sided. *P* < 0.05 were considered statistically significant. The statistical analysis was performed using GraphPad Prism 5.

## Results

### Serum IgG4 in the Differential Diagnosis of CP

**Figure 1:**
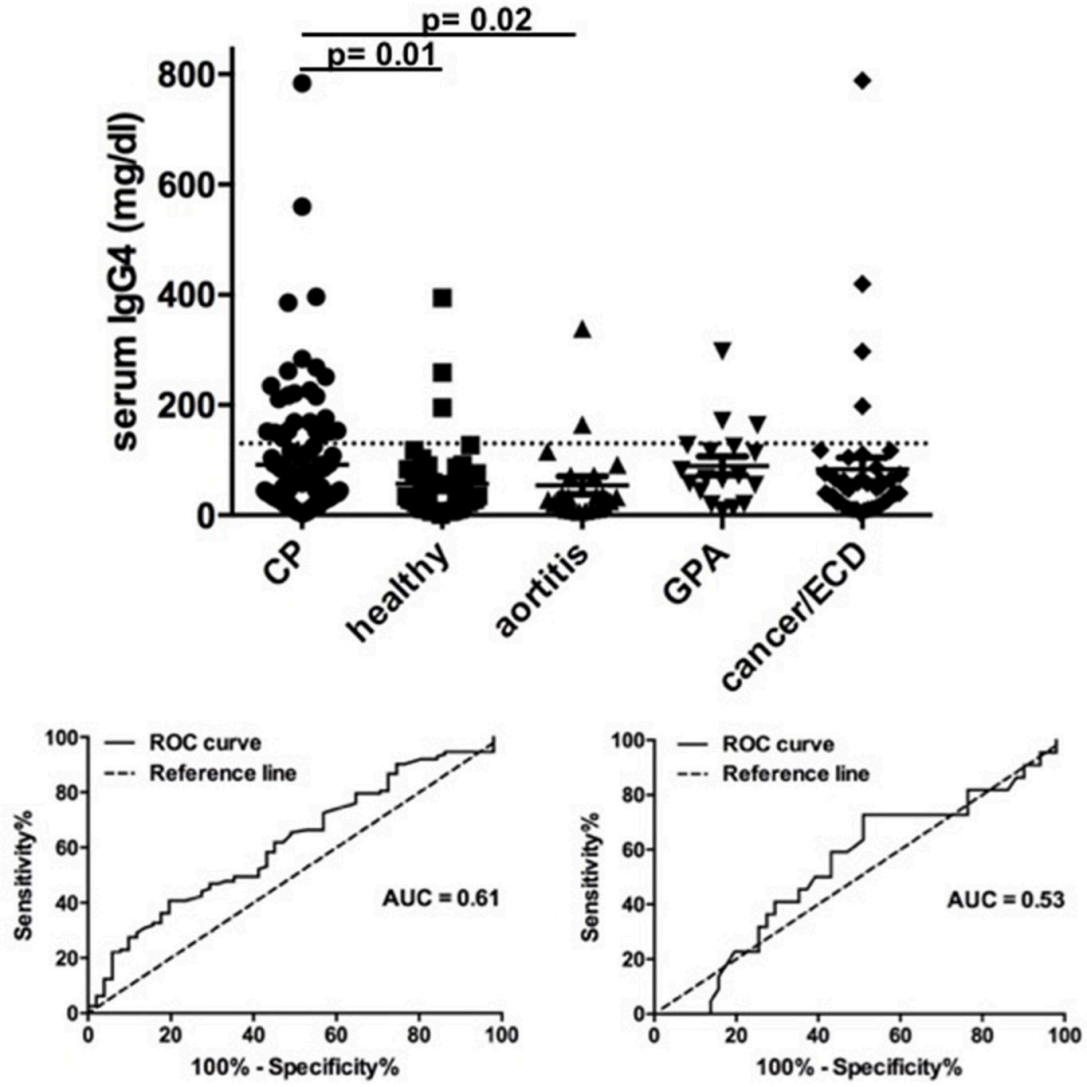
Serum IgG4 levels in chronic periaortitis patients and controls. In the upper figure, the plot shows serum IgG4 levels in patients with active chronic periaortitis, healthy controls, patients with aortitis (secondary to Takayasu arteritis or giant-cell arteritis), granulomatosis with polyangiitis (GPA, Wegener's) and retroperitoneal malignancies or Erdheim-Chester disease (ECD). The horizontal line indicates the upper limit of normal of serum IgG4 (135 mg/dL). In the lower figures, the receiver operating characteristic (ROC) curves show the sensitivity and specificity of serum IgG4 in discriminating chronic periaortitis patients from healthy controls **(left)** and aortitis patients **(right)**.

One hundred and thirteen CP patients were included in the study. Twenty-four patients (21.2%) had high serum IgG4 (>135 mg/dL) and their median serum IgG4 level was 216 mg/dL (IQR 153–263.5). In addition to CP patients, 51 healthy controls, 41 patients with retroperitoneal malignancies or ECD, 22 patients with aortitis (secondary to Takayasu or giant cell arteritis), and 18 with GPA were tested for serum IgG4; all patients were tested at the time of diagnosis, before the start of treatment. Serum IgG4 levels were significantly higher in CP patients than in healthy controls (*p* = 0.01) and in CP patients than in patients with aortitis (*p* = 0.02) (Figure 1). No statistically significant differences were observed between CP patients and patients with neoplasms/ECD (*p* = 0.21) or GPA (*p* = 0.42). Receiver operating characteristic (ROC) analysis of the sensitivity and specificity of IgG4 in discriminating CP vs. healthy controls or aortitis patients showed poor reliability of this marker (Figure 1).

### Baseline Characteristics

The main demographic and clinical characteristics of the patients at disease onset are reported in Table 1. The proportion of men was higher in the group of patients with high serum IgG4 compared to that with normal IgG4 (83 vs. 64%) but the difference was of borderline statistical significance (*p* = 0.09). The two groups did not differ in terms of comorbidities (cardiovascular or autoimmune diseases) or presenting signs or symptoms (Table 1); the rates of disease-related complications (e.g., hydronephrosis, acute renal failure, deep vein thrombosis) were also comparable between the two groups. The characteristics of CP on CT and MRI were also similar: in particular, there were no statistically significant differences between the two groups in terms of proportion of aneurysmal forms, atypical localization (e.g., pelvic, isolated periureteral) and involvement of the thoracic vessels and/or mediastinum (i.e., thoracic periaortitis, fibrosing mediastinitis). A similar maximal CP thickness at baseline (on CT or MRI) was also found. In addition, the maximal mass thickness did not correlate with serum IgG4 levels (ρ = 0.13; *p* = 0.19). The main laboratory parameters were also similar between the two groups. Baseline serum IgG4 levels did not correlate with ESR, CRP, serum creatinine, hemoglobin, and leukocyte count (data not shown).

**Table 1:** Main characteristics of the 113 patients at the time of inclusion in the study.

	**All patients**	**Serum IgG4 level**		***p-*value**
		**Normal**	**High (>135 mg/dL)**	
**NO. OF PATIENTS**, ***n (%)***	113	89 (78.8)	24 (21.2)	
Age, years–*median (IQR)*	57 (51–66)	57 (50–64)	59 (52.7–62.7)	0.27
Male gender, *n (%)*	77 (68.1)	57 (64.0)	20 (83.3)	0.09
**COMORBIDITY, *n* (%)**				
Cardiovascular disease^*^	29 (25.7)	24 (26.9)	5 (20.8)	0.61
Autoimmune disease	39 (34.5)	31 (34.8)	8 (33.3)	1.00
Autoimmune thyroiditis	24 (21.2)	16 (17.9)	3 (12.5)	0.76
Other autoimmune diseases	20 (17.7)	19 (21.3)	6 (25.0)	0.78
**CLINICAL MANIFESTATIONS, *n* (%)**				
Constitutional symptoms	75 (66.4)	59 (66.3)	16 (66.7)	0.62
Abdominal/lumbar pain	91 (80.5)	71 (79.7)	20 (83.3)	0.78
Deep vein thrombosis	11 (9.7)	8 (8.9)	3 (12.5)	0.69
Hydronephrosis	81 (71.7)	66 (74.2)	16 (66.7)	0.45
Unilateral	35 (30.9)	29 (32.6)	6 (25.0)	0.62
Bilateral	46 (40.7)	37 (41.6)	10 (41.7)	1.00
Acute renal failure	52 (46.0)	43 (48.3)	9 (37.5)	0.37
Extra-retroperitoneal fibro-inflammatory lesions^§^	16 (14.1)	8 (8.9)	8 (33.3)	0.005
**LABORATORY MARKERS**				
ESR, mm/h–*median (IQR)*	53 (39–77.5)	53 (38–74)	61 (39–90)	0.45
CRP, mg/L–*median (IQR)*	14.1 (5.4–31.8)	13.0 (5.4–31.6)	19.5 (5.8–32.1)	0.51
WBC, × 10^9^-*median (IQR)*	7.4 (6.1–8.7)	7.4 (6.2–8.8)	6.9 (5.7–7.9)	0.28
Hemoglobin, g/dl–*median (IQR)*	12.4 (11.1–13.5)	12.5 (11.2–13.7)	11.4 (10.8–13.4)	0.22
Creatinine, mg/dl– *median (IQR)*	1.3 (0.9–3.4)	1.3 (0.9–3.5)	1.3 (1.0–3.2)	0.49
ANA positivity, *n (%)*	27 (23.9)	21 (23.6)	6 (25.0)	1.00
IgG4, mg/dL–*median (IQR)*	51 (28–110)	41 (20–71)	216 (153–263.5)	0.0001
**CP CHARACTERISTIC**				
Perianeurysmal RPF, *n (%)*	14 (12.4)	11 (12.3)	3 (12.5)	1.00
Atypical localization^¶^, *n (%)*	13 (11.5)	9 (10.1)	4 (16.7)	0.47
Thoracic vessel involvement, *n (%)*	20 (17.7)	15 (16.8)	5 (20.8)	0.76
Maximal mass thickness, mm–*median (IQR)*	15 (10.0–22.0)	15 (10.5–20.0)	18 (10.5–23.8)	0.26
**^18^F-FDG UPTAKE AT BASELINE^#^**				
	*n = 60*	*n = 47*	*n = 13*	
Grade 0, *n (%)*	6 (10)	6 (12.7)	0 (0)	0.32
Grade 1, *n (%)*	3 (5)	2 (4.3)	1 (7.7)	0.52
Grade 2, *n (%)*	17 (28.3)	13 (27.7)	4 (30.8)	1.00
Grade 3, *n (%)*	34 (56.7)	26 (55.3)	8 (61.5)	0.76

*IQR, interquartile range; ESR, erythrocyte sedimentation rate; CRP, C-reactive protein; WBC, white blood cell; ANA, anti-nuclear antibodies; CP, chronic periaortitis; RPF, retroperitoneal fibrosis; FDG, fluorodeoxyglucose*.

* *Cardiovascular disease denotes overt ischemic heart disease, cerebrovascular or peripheral arterial disease*.

§ *Extra-retroperitoneal fibro-inflammatory lesions denote lesions that can be included in the spectrum of IgG4-related disease, such as mediastinal fibrosis, thoracic periaortitis, chronic sclerosing pancreatitis, renal disease (e.g., membranous nephropathy, tubule-interstitial nephritis) (see text for details)*.

¶ *Atypical localization denotes pelvic, isolated peri-ureteral, and other localizations of retroperitoneal fibrosis other than the typical peri-aortoiliac one*.

# *FDG PET was performed at baseline (i.e., at the time of baseline IgG4 measurement, during active disease) in 60 of the 113 chronic periaortitis patients included in the study*.

**Figure 2:**
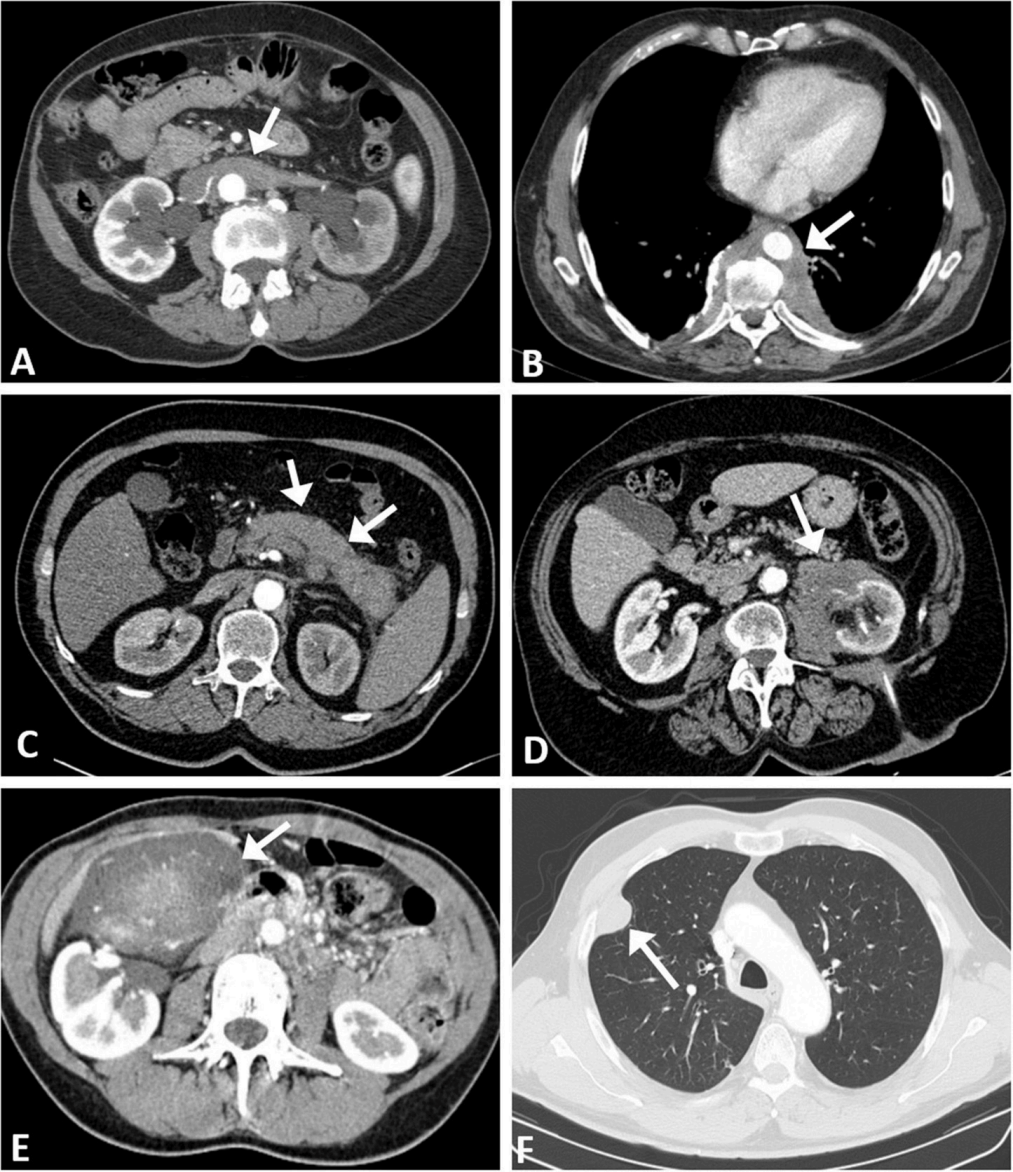
CT Imaging overview of chronic periaortitis and extra-retroperitoneal lesions. **(A)** Sleeve thickening of the abdominal aorta (*arrow*) with involvement of the renal arteries; the fibro-inflammatory tissue wraps around the excretory system and determines bilateral hydronephrosis. **(B)** Typical aspect of “coated aorta” involvement along the descending thoracic tract: the inflammatory tissue (*arrow*) also expands into the paravertebral space and envelops the verterbal bodies. **(C)** IgG4-related chronic pancreatitis: the normal glandular structure is subverted (*arrows*) and a pseudonodular lesion is evident at the level of the pancreatic tail. **(D)** Atypical localization of retroperitoneal fibrosis: the pathological tissue encroaches into the left perirenal space (*arrow*) without involvement of excretory system. **(E)** Unusual gallbladder localization of IgG4-related disease: a bulky mass (*arrow*) attached to the bottom of the gallbladder with large cholecystic feeding vessels. **(F)** Thoracic localization: a pseudonodular lesion arises from the right pleural sheet (*arrow*).

Patients with high serum IgG4 at baseline had a higher incidence of extra-retroperitoneal fibro-inflammatory lesions (namely lung and pleural disease compatible with IgG4-related pulmonary involvement, fibrosing mediastinitis, membranous nephropathy, cholecystitis, chronic sialoadenitis, and autoimmune pancreatitis) compared to patients with normal serum IgG4 (33.3 vs. 8.9%, *p* = 0.005) (Table 1, Figure 2). None of these lesions- taken individually- drove the association with an increased IgG4 level.

Sixty patients (53%) had undergone FDG-PET at baseline, before the start of treatment; of them, 47 (78%) had normal serum IgG4 levels and 13 (22%) had high levels. No differences were found in the ^18^F-FDG uptake grade between patients with normal and high IgG4 (Table 1). In addition, there was no statistically significant correlation between the maximum standardized uptake value (SUVmax) at baseline and serum IgG4 levels (ρ = −0.28; *p* = 0.09).

### Treatment and Follow-Up

Data about treatment and post-treatment follow-up were available for 104 patients, of whom 84 (80.8%) had normal serum IgG4 and 20 (19.2%) had high IgG4 at baseline (Table 2). Sixty-one patients (58.6%) had received induction therapy with prednisone (initial dose, 1 mg/kg/day for the first month, progressively tapered during the ensuing 6–9 months). Six patients (5.8%) had been treated with prednisone for 1 month followed by tamoxifen (fixed dose of 0.5 mg/kg/daily) for 8 months. Thirty-seven patients (35.6%) had received prednisone (initial dose, 0.8–0.5 mg/kg/daily) and an immunosuppressive agent (i.e., mycophenolate mofetil, methotrexate) for at least 9 months. The distribution of the different therapeutic regimens was comparable between patients with normal and high serum IgG4 (Table 2).

**Table 2:** Main treatments and outcome of the chronic periaortitis patients included in the study.

	**All patients**	**Serum IgG4 level at baseline**		***P*-value**
		**Normal**	**High**	
NO. OF PATIENTS^*^	104	84	20	
**TREATMENT REGIMEN**				
PDN, *n (%)*	61 (58.6)	51 (60.8)	10 (50.0)	0.45
PDN and TXF, *n (%)*	6 (5.8)	5 (5.9)	1 (5.0)	1.00
PDN and IS, *n (%)*	37 (35.6)	28 (33.3)	9 (45.0)	0.43
**OUTCOME**				
Remission, *n (%)*	90 (86.5)	72 (85.7)	18 (90.0)	1.00
% Reduction in CP thickness, *median (IQR)*	50 (27.4–66.6)	50 (27.3–66.7)	50 (27.7–67.5)	0.26
Relapses, *n (%)*	35/83 (42.2)	29/65 (44.6)	6/18 (33.3)	0.78
Follow-up duration, months, *median (IQR)*	36 (16–67)	33 (15.5–67.7)	39 (18–55)	0.85

* *Only 104 of the 113 patients studied had available follow-up data and were therefore included in this analysis*.

*PDN, prednisone; TXF, tamoxifen; IS, immunosuppressants; CP, chronic periaortitis; IQR, interquartile range*.

Ninety patients (86.5%) achieved remission following treatment. The percentage of patients who achieved remission was similar between the groups with normal and high IgG4 levels (85.7 and 90.0%, respectively, *p* = 1.00). In addition, the median percent change in the mass thickness on follow-up CT or MRI (month 9 of treatment vs. baseline) did not differ between these two groups of patients (Table 2). Serum IgG4 levels did not correlate with the CT/MRI-documented mass thickness reduction (ρ = 0.05; *p* = 0.63).

**Figure 3:**
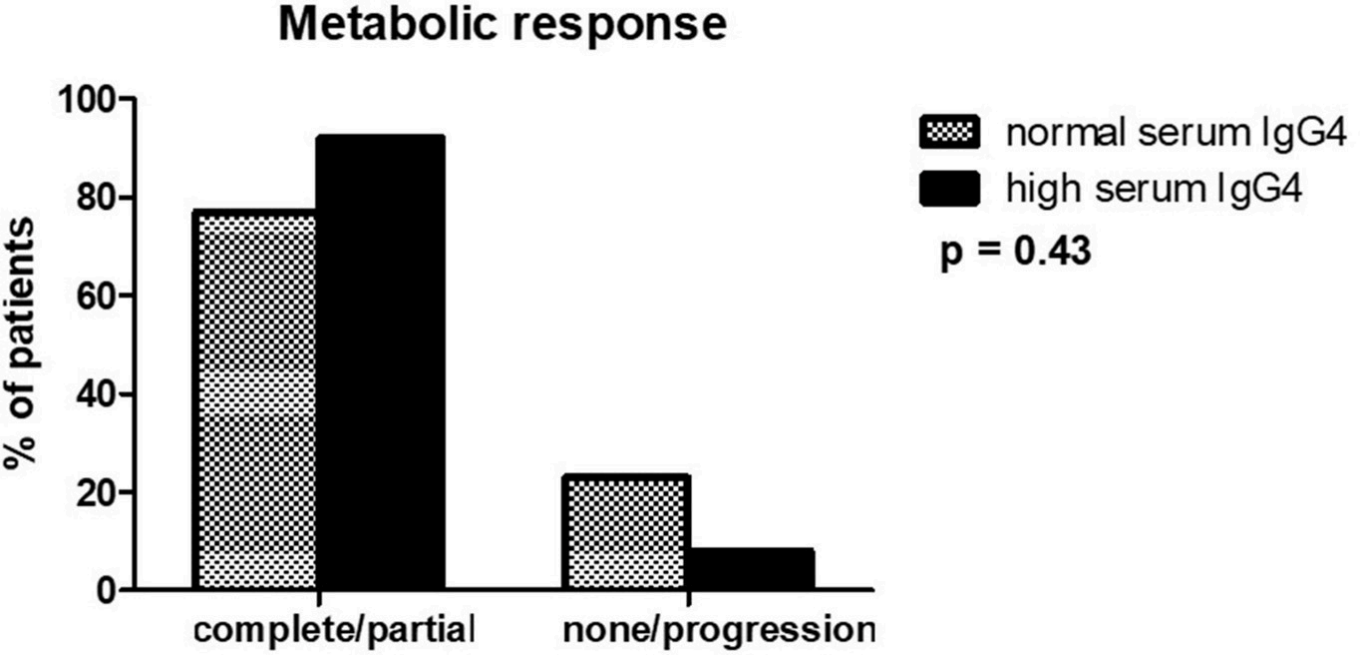
Metabolic response to treatment assessed by FDG-PET in patients with high and normal serum IgG4 levels. Responses were evaluated as described in the text (see Methods section).

**Figure 4:**
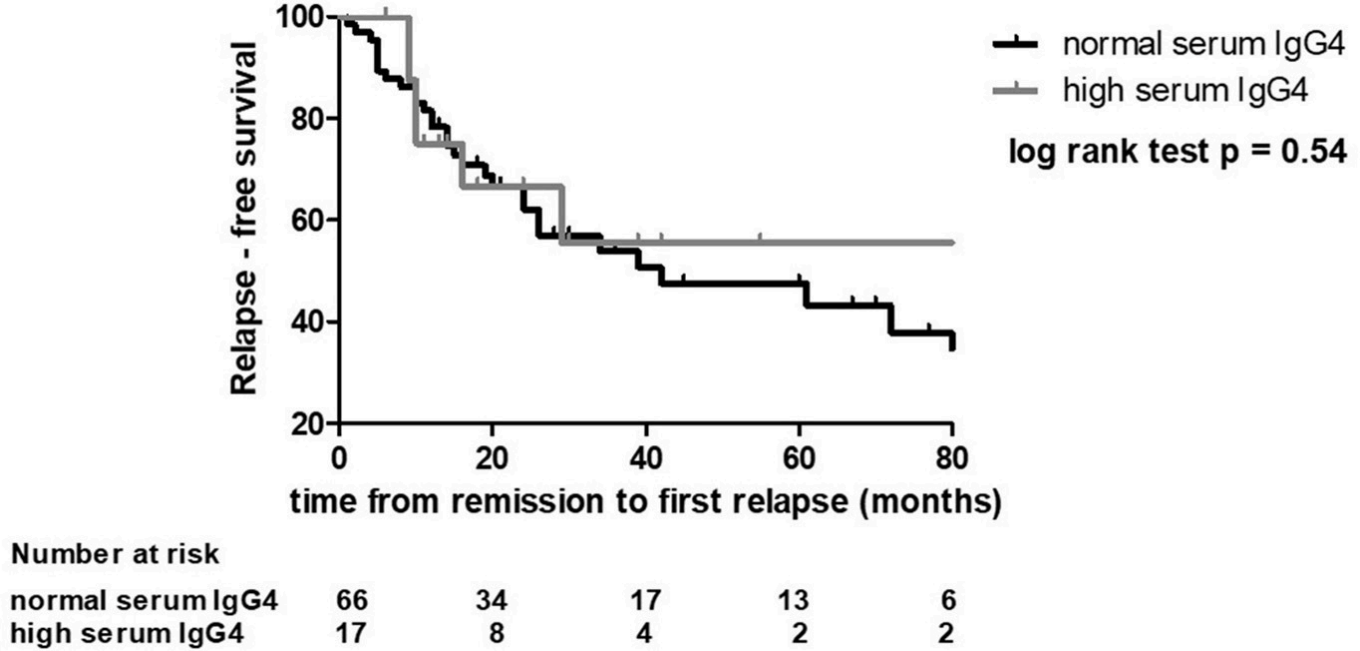
Relapse-free survival in patients with high and normal serum IgG4 levels. Relapse-free survival was the time from remission to relapse or last follow-up.

All the 60 patients who had undergone FDG-PET at baseline repeated it at the end of treatment. We did not find any differences in the rates of metabolic responses between the high- and normal-IgG4 groups (Figure 3). After remission, seven patients were lost to follow-up. The median follow-up after remission of the remaining 83 patients was 36 months (IQR 16–67). During this period, 29 patients with normal serum IgG4 and 6 patients with elevated serum IgG4 at baseline had a relapse (Table 2). The median time to relapse was 14 months (IQR 5.5–26) in the first group and 10 months (IQR 9.3–14.5) in the second group (log-rank test *p* = 0.54) (Figure 4). The presence of extra-retroperitoneal lesions did not affect the probability to achieve remission or to develop relapse (data not shown).

### Pathology Findings

Retroperitoneal tissue samples were available from 23 patients, of whom 18 had normal serum IgG4 levels and five high levels. Nineteen biopsies (82.6%) exhibited an active inflammatory disease, with dense lymphoplasmacytic infiltrates admixed with fibrosis, while in four (17.4%) the inflammatory infiltrate was almost absent and fibrosis dominated. The median number of IgG+ plasma cells and of IgG4+ plasma cells infiltrating the tissue did not significantly differ between patients with normal or high serum IgG4, although it tended to be higher in the latter group (*p* = 0.08). Only two patients (8.7%) had a tissue IgG4+/IgG+ plasma cell ratio >40%. One of them had high serum IgG4 levels and also extra-retroperitoneal localization of IgG4-RD (thyroiditis). The other one had normal serum IgG4 levels and no extra-retroperitoneal disease localizations.

No statistically significant correlations were found between serum IgG4 levels and plasma cell tissue infiltration (CD138+ cells) or between serum IgG4 levels and the IgG4+/IgG+ plasma cell ratio in the tissue (ρ = 0.10; *p* = 0.67 and ρ = 0.27; *p* = 0.26, respectively), probably because of the small number of biopsies analyzed.

## Discussion

CP is a rare condition with a benign course if promptly recognized and treated (1, 19). However, its diagnosis often represents a challenge for the clinicians, as it requires the exclusion of several etiologies. CP has recently been included in the spectrum of IgG4-RD together with other fibro-inflammatory disorders involving different organs or systems, which reinforces the view that CP may be a manifestation of a systemic immune-mediated condition (5, 16). The introduction of the concept of IgG4-RD has led to the question as to whether IgG4-related and—unrelated forms of each given lesion included under this umbrella differ in terms of clinical presentation, response to treatment, and prognosis. This has also been explored in patients with CP, with conflicting results: while some authors reported that IgG4-related CP cases are more inflammatory (23), others showed that they are less symptomatic (and therefore, presumably less inflammatory) (24). In terms of outcome, higher relapse rates were reported in IgG4-related CP patients (14), but this was not further confirmed (23). The only consistent finding across the studies is the higher incidence of extra-retroperitoneal manifestations in the IgG4-related subgroup (10, 12, 25). The studies performed so far, however, are often limited by small samples (they included up to 59 cases) and the frequent lack of data regarding response to treatment and long-term outcome (10, 12–16, 23, 25, 26).

In our study, performed on a cohort of 113 consecutive CP patients with a median follow-up >3 years, we demonstrate that the clinical phenotype and outcome of IgG4-related and—unrelated CP (based on IgG4 serum levels) is quite similar. When we compared the clinical and laboratory characteristics of the two subgroups at presentation, we found analogous frequencies of disease-related symptoms, hydronephrosis, and acute kidney failure. Likewise, the levels of acute-phase reactants such as ESR and CRP were comparable, and so was the frequency of positive anti-nuclear antibodies and associated autoimmune diseases. In keeping with previous studies (10, 26), we also observed similar imaging findings on CT/MRI between IgG4-related and—unrelated CP, including the maximal thickness of the periaortic tissue and the proportions of patients with aneurysmal forms or with involvement of the thoracic aorta. This latter finding is in line with a previous study of ours that showed comparable IgG4 levels between patients with localized abdominal CP and patients with diffuse (abdominal plus thoracic) CP (27). More than half of our patients underwent FDG-PET at the time of active disease, while they were untreated. No differences were detected in the grade of FDG uptake between patients with high and normal IgG4. FDG-PET is considered a reliable metabolic modality to assess the inflammatory activity of CP (22, 28–30); therefore, the similar FDG uptake at presentation in the two subgroups indicates- together with the comparable levels of ESR and CRP- that the degree of inflammation in CP patients at presentation is not influenced by the IgG4 status.

The only phenotypic difference we observed with respect to the IgG4 status lies in the more frequent occurrence of extra-retroperitoneal fibro-inflammatory lesions in the high IgG4subgroup, thus suggesting that patients with a more pronounced IgG4 response are more likely to have a systemic disease than those with normal levels, as previously demonstrated (10, 12, 14–16).

We also evaluated response to treatment and relapses in the two subgroups. Most of our patients had received conventional therapies based on glucocorticoids, alone or in combination with immunosuppressants, with no significant differences in the distribution of these treatment regimens between the IgG4-related and—unrelated groups. Response to therapy was assessed as percentage of patients achieving remission (with remission being a composite end-point of disappearance of disease-related manifestations, normalization of acute-phase reactants, and resolution of hydronephrosis) (19) as well as reduction in maximal thickness of the periaortic mass and metabolic responses on FDG-PET. All of these outcome measures were comparable between the two groups; likewise, the probability of relapse, assessed as relapse-free survival, was similar. Patient outcome was seldom assessed by previous studies; our results clearly indicate that the IgG4 status is not a predictor of either response or relapse in CP patients, and that IgG4 levels should not condition treatment strategies. Rituximab is a valuable treatment option in IgG4-RD. Four of our patients received Rituximab: all of them had normal IgG4 serum levels and all achieved remission. This finding, although preliminary, suggests that also IgG4-unrelated lesions (with IgG4-relatedness being based on serum IgG4 levels)—and not only IgG4-related forms- may be sensitive to this drug.

We classified patients with CP into IgG4-related and—unrelated based on serum IgG4 levels rather than on tissue evidence of IgG4-plasma cell infiltration. This is remarkably different from most of the previous studies, and it must be acknowledged that up to 40–50% of patients with biopsy-proven IgG4-RD have normal serum IgG4 (31–34). The analysis of the 23 biopsies available from our patients confirms that there is not always a correlation between tissue IgG4+/IgG+ plasma cell ratio and serum IgG4 levels. The paucity of histological data is certainly a limitation of our work, but it must be considered that diagnostic biopsies are not routinely performed in CP, and therefore the clinical significance of non-invasive biomarkers such as serum IgG4 needs to be clarified. However, our data appear to confirm that serum IgG4 levels cannot be considered a good biomarker of IgG4-RD, particularly of patients with (isolated) CP. In our study we also showed that serum IgG4 levels do not help in the differential diagnosis between CP and other conditions such as aortitis, ECD, and malignancies.

We observed that only 21% of CP patients had high serum IgG4, which is lower than the percentage reported by other authors (usually ranging between 28 and 60%) (10, 23, 25, 26); this discrepancy may be accounted for by several reasons, such as ethnic differences or referral bias (other centers may receive patients with more systemic forms- which are more frequently IgG4-positive, while our center is primarily a CP referral center). However, our patients were consecutive, and IgG4 levels were always measured at the time of active disease, before any treatment was started.

Despite these limitations of our study, and also considering its retrospective nature, we believe that the data we provide are quite solid, and that the large size of our cohort, the long follow-up, the accurate assessment of clinical presentation and outcomes are important strengths. It appears therefore that IgG4 cannot help differentiate subsets of patients with different disease severity or prognosis, thus new biomarkers are warranted. Certainly, based on our findings, treatment or follow-up strategies should not be tailored on the IgG4 status of CP patients.

In conclusion, the clinical phenotype, inflammatory activity, response to treatment, and relapse probability of CP patients with high or normal serum IgG4 levels are comparable. Patients with high IgG4 levels are more likely to have multisystemic involvement, and should therefore be investigated more carefully for the occurrence of extra-retroperitoneal lesions.

## Data Availability

The datasets generated for this study are available on request to the corresponding author.

## Author Contributions

FM: study design, data collection and analysis, manuscript drafting; RR, EA, CM, MU, PF, GB, and AP: data collection; MN and DC: data collection (histological analysis); FP: statistical analysis; AAP: radiological evaluation- data collection; GE: study design, data collection; NP: data collection, manuscript drafting; AV: study design, data collection and analysis, manuscript writing.

### Conflict of Interest Statement

The authors declare that the research was conducted in the absence of any commercial or financial relationships that could be construed as a potential conflict of interest.
